# Delivery of Biomolecules into Individual Cells and Subcellular Compartments by Localized Electroporation via Nanopipette

**DOI:** 10.1021/acsnanoscienceau.5c00053

**Published:** 2025-08-01

**Authors:** Fabio Marcuccio, Philip S. Goff, Devkee M. Vadukul, Fawaz Raja, Yilin Li, Ren Ren, Debjani Saha, Luca Magnani, Francesco A. Aprile, Uma Anand, Elena V. Sviderskaya, Joshua B. Edel, Aleksandar P. Ivanov, Petr V. Gorelkin, Yuri Korchev, Andrew Shevchuk

**Affiliations:** † Faculty of Medicine, 4615 Imperial College London, Hammersmith Campus, Du Cane Road, London W12 0NN, U.K.; ‡ Genomics Research Centre, Human Technopole, Viale Rita Levi-Montalcini 1, Milan 20157, Italy; § School of Health and Medical Sciences, City St George’s, 200477 University of London, Cranmer Terrace, London SW17 0RE, U.K.; ∥ Department of Chemistry, 683262 Imperial College London, Molecular Sciences Research Hub, White City Campus, 82 Wood Lane, London W12 0BZ, U.K.; ⊥ The Breast Cancer Now Toby Robins Research Centre, The Institute of Cancer Research, 123 Old Brompton Road, London SW7 3RP, U.K.; # ICAPPIC Limited, London NW10 6TD, U.K.; ∇ WPI Nano Life Science Institute (WPI-NanoLSI), Kanazawa University, Kanazawa 920-1192, Japan

**Keywords:** Injection, nanoinjection, cell delivery, single-cell, electroporation, SICM, scanning-probe microscopy

## Abstract

Introducing exogenous biomolecules into individual cells with precise control over space, time, and dosage is crucial for both fundamental and applied biological research. Glass nanopipettes have long been employed to deliver biomolecules into individual cells; yet, their reliance on the electrical charge of the target molecule and the need for penetrating the cellular membrane pose significant limitations. We demonstrate that voltage pulses applied through a glass nanopipette in proximity to the cell membrane induce localized electroporation and generate directional flow, enabling controlled delivery of both charged and neutral biomolecules into subcellular compartments, e.g., the nucleus, without the need for penetrating the cellular membrane. This approach minimizes cell damage and preserves cell viability, even after multiple rounds of injection. Our findings will serve as a reference for the design of novel nanopipette methods, contributing to the newly established field of spatiotemporal analysis of live cells.

## Graphical abstract

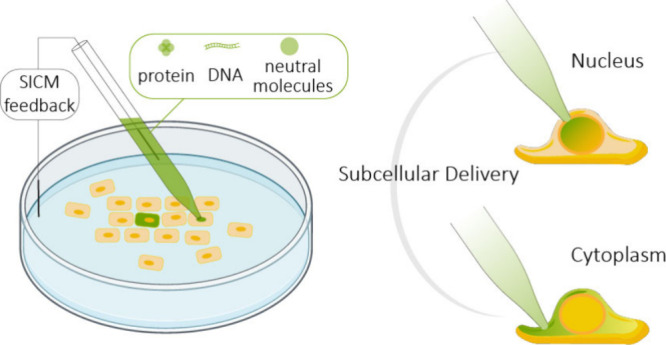

## Introduction

With growing interest in spatiotemporal analysis of live cells,^1,2^ there is an increasing need to develop techniques capable of perturbing individual cells within their physiological environment. Effective perturbations rely critically on the successful delivery of exogenous molecules into individual cells with tight control over the time, location, and dosage of the delivery.

Several techniques have been developed to address molecular delivery into cells. Fluidic Force Microscopy (FluidFM) integrates microfluidics with atomic force microscopy to enable precise manipulation and injection at the single-cell level,^3–5^ but requires physical insertion of the probe within the cellular compartments, disrupting the actin cytoskeleton. Vertical nanoneedles have been used for high-throughput intracellular delivery of nucleic acids and proteins,^6–8^ but they do not allow single-cell delivery. Porous silicon-mediated optoporation has been used for spatially resolved transfection of mRNA in organoids, but rely on nanoparticle functionalization and nonspecific interactions between cells and nanoparticles.^9^

Glass nanopipettes, with tips ranging from ∼10 to hundreds of nanometers, are used to deliver molecules into cells with minimal disruption compared to traditional microinjection.^10–12^ Unlike pressure-driven microinjection, nanoinjection relies on electrophoresis to control molecule delivery based on applied voltage and molecule charge.^13–18^ Recent advances include combining nanopipettes with electroporation,^19,20^ however the underlying transport mechanism, attributed solely to diffusion, remains elusive.

Beyond injection, nanopipettes enabled manipulation of individual cells, including cytoplasmic biopsies,^18,21–23^ mapping of subcellular compartments,^24^ pH mapping,^25,26^ measurement of reactive oxygen/nitrogen species,^27–29^ and biosensing.^30–33^ Furthermore, nanopipettes can be used as probes for scanning ion-conductance microscopy (SICM), a noncontact scanning probe technique for topographical mapping of live cells in solution. SICM has enabled a wide range of applications,^18,24–26,34–39^ including single-molecule injection.^17^

In glass nanopipettes, the ion current arises from the transport of ionic species under the influence of an applied electric field. This process is governed by a combination of electrophoresis, electroosmosis, and ionic concentration gradients, making the underlying physics significantly more complex.^40–43^ By adjusting the relative contributions of electroosmotic and electrophoretic flows, it is possible to control the movement of ions or molecules through the nanopipette, depending on their physicochemical properties, and direct them in or out of the tip.^13,17,37^ Furthermore, integration with SICM and electroporation offers precise spatial control, enabling the targeted delivery of biomolecules without the need for penetration into the cell.

We present a novel method for delivering exogenous molecules into individual cells using glass nanopipettes, overcoming limitations of traditional techniques. By applying voltage to the inner electrode of a nanopipette, both liquid and electrophoretic flows are generated, allowing the ejection of neutral and charged molecules, respectively. When integrated with SICM and single-cell electroporation, this method allows for the precise delivery of biomolecules into specific cellular compartments (i.e., nucleus or cytoplasm) without damaging the cellular membrane and with high spatial and temporal resolution. Finally, we highlight the importance of tailoring the electroporation pulse shape and polarity according to the physicochemical properties of the biomolecule of interest. Not all biomolecules respond uniformly to the same pulse program, and inappropriate experimental designs can lead to suboptimal or unexpected results.

## Results

### Voltage-Induced Liquid Flow within a Nanopipette

We designed an electrochemical nanoprobe to investigate whether the application of a voltage to the inner electrode of a nanopipette generates electrophoretic flow that results in directional bulk liquid flow. The direction of the bulk liquid flow that can carry neutral molecules is determined by the direction of the electrophoretic flow of ions with higher hydration number, while the magnitude of the flow depends on the ionic strength of the nanopipette inner solution, the applied voltage, and the size of the nanopipette aperture. The nanoprobe consisted of a double barrel nanopipette fabricated from quartz capillaries (Figure 1A). Pyrolytic carbon was deposited in one barrel (carbon barrel) following established methods.^38,44–46^ The pyrolytic carbon serves as an electrode for electrochemical measurements and is connected to the amplifier via an Ag wire. The second barrel (open barrel) was filled with 1 mM redox mediator in 100 mM KCl and contained an Ag/AgCl electrode for ion current measurements (Electrical resistance R = 27*M*Ω measured with 100 mM KCl pipette and bath solution). The back end of the open barrel was also connected to a pressure pump equipped with a closed-loop controller, enabling the accurate application of pressure. During the experiment, the nanoprobe was immersed in a bath containing 100 mM KCl. The characterization of the nanoprobe with the cyclic voltammetry performed at the carbon electrode and open barrel can be found in the Supporting Information, Section 2.1, Figure SF1.

**Figure 1:**
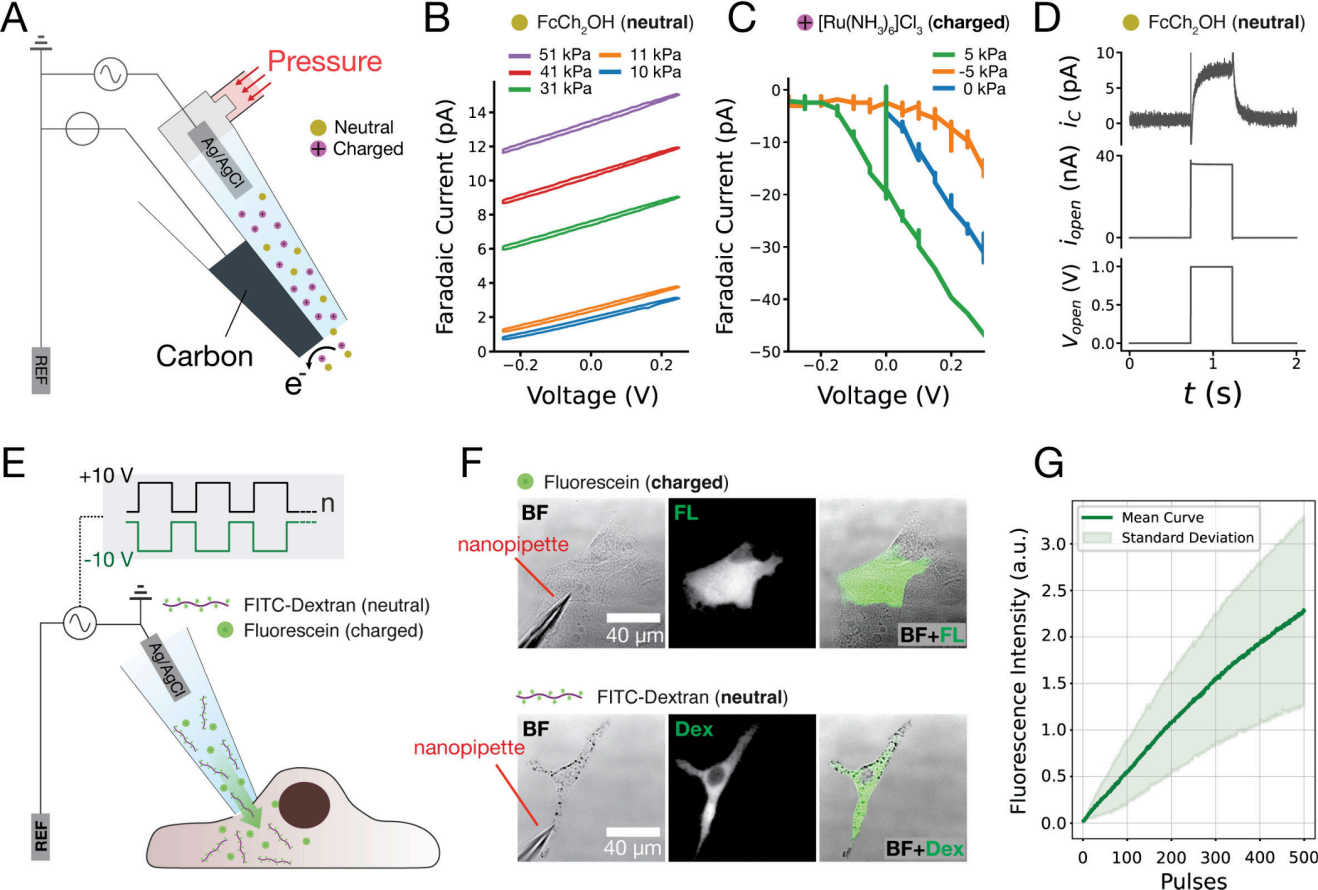
Voltage-induced flow integration with single-cell electroporation. (A) Double-barrel nanopipette used in the experiment. One barrel contains pyrolytic carbon, while the other contains an electrolyte solution with an electrically neutral or charged mediator and an Ag/AgCl electrode. The second barrel is connected to a pressure control. The nanoprobe is immersed in a bath containing the same solution as the open barrel without the electric mediator. (B and C) Faradaic current measured at the carbon electrode held at +300 mV under different pressure and voltage conditions in the case of a neutral (B) and charged (C) mediator. (D) Faradaic current (i_C_) detected at the carbon electrode, ion current detected at the Ag/AgCl electrode (i_open_) under the application of a 500 ms, 1 V voltage pulse (V_open_). (E) Single-cell electroporation setup. A glass nanopipette containing the molecule of interest (charged or neutral) is brought in proximity to the cell membrane and a train of voltage pulses is delivered to achieve temporal permeabilization of the membrane and delivery of biomolecules within the cell. (F) Example of a delivery of FITC (charged) and FITC-dextran (neutral) molecules within a human breast cancer cell line (MCF7) and immortal mouse melanocytes (melan-a). (G) Real time fluorescence intensity of FITC-Dextran detected at a confocal microscope during electroporation of cells. *N* = 10.

We reasoned that applied potential at the Ag/AgCl electrode in the open barrel would generate bulk flow and carry neutral mediator molecules out of the barrel, enabling their reduction or oxidation at the carbon electrode. In the first experiment, we used ferrocenemethanol (*FcCH*_2_ *OH*) as a redox mediator. In this organometallic compound, an iron ion is sandwiched between two cyclopentadienyl ligands, each donating a single negative charge balancing the iron center’s positive charge, resulting in charge neutrality. When a pressure was applied using the pressure pump, we observed a linear curve (R^2^ = 0.99, Supporting Information, Section 2.2, Figure SF2) increase in the faradaic current at the carbon electrode (held at +300 mV) as the mediator was ejected from the open barrel and underwent redox reactions (Figure 1B). This linear relationship confirmed that stronger applied pressure resulted in a greater number of mediator molecules reacting at the carbon electrode. Interestingly, we also observed a linear change in the faradaic current detected at the carbon electrode by sweeping the applied electric potential at the Ag/AgCl electrode in the open barrel from −0.25 V to +0.25 V under constant pressure applied by the pump. Given the charge neutrality of the mediator, we attributed this phenomenon to a voltage-induced bulk flow of liquid that was added to or subtracted from the flow created by the pressure generated by the pump, depending on voltage polarity. The characterization of voltage-induced liquid flow was also performed by applying a ramp and steps of potential in the presence and absence of a mediator in the open barrel, and results can be found in Supporting Information, Section 2.3, Figure SF3. We repeated the experiment using a solution of 1 mM hexa ammineruthenium­(III) chloride ([Ru­(NH_3_)_6_]­Cl_3_) as the mediator in 100 mM KCl in the open barrel. Unlike ferrocenemethanol, the ruthenium ion (*Ru* ^*3+*^) carries a strong positive charge due to its +3 oxidation state. Figure 1C shows the faradaic current measured at the carbon electrode while applying different pressure and voltage magnitudes to the Ag/AgCl electrode in the open barrel (Supporting Information, Section 2.4, Figure SF4). Interestingly, the contributions of voltage polarity and magnitude to the faradaic current are more pronounced compared to the case of the neutral mediator. At negative potentials, no faradaic current was detected as the charged mediator molecules migrated toward the negative electrode and remained within the barrel. Conversely, positive potentials resulted in a significant faradaic current, as the mediator molecules were repelled by the nanopipette electrode and ejected from the barrel to react at the carbon electrode. When pressure was applied, the faradaic current did not change as much as observed in the case of neutral mediator (Figure 1B). This is due to the electrophoretic flow having a greater contribution to the transport of charged molecules compared to the pressure-driven liquid flow. Next, we checked whether flow bursts could also be generated by applying short high-magnitude voltage pulses at the Ag/AgCl electrode in the case of ferrocenemethanol solution in the open barrel. The application of a 500 ms pulse with 1 V magnitude generated ∼7–8 pA faradaic current at the carbon electrode (Figure 1D), corresponding to flow-generated mechanical pressure of ∼29–33 kPa (Supporting Information, Section 2.2, Figure SF2). The magnitude of the generated liquid flow is influenced by the diameter of the nanopipette aperture and geometry, but under high voltage biases (e.g., 10 V), moderate variations in the pore diameter do not significantly affect the resulting liquid flow.^41^ We conclude that electric potential generates an electrophoretic flow, which in turn results into a liquid flow that enables the ejection of both charged and neutral molecules. For charged species, electrophoretic mobility is the dominant transport mechanism, whereas neutral molecules are transported primarily by voltage-induced bulk flow. This ability offers a versatile strategy for controlled delivery, while the flow bursts generated under the application of potential pulses make it suitable for integration into single-cell electroporation systems.

### Single-Cell Electroporation with Voltage-Induced Flow Bursts

We used a single-barrel glass nanopipette (Electrical resistance R = 50*M*Ω measured with 100 mM KCl pipette and bath solution, corresponding to an estimated pore diameter of ∼ 160 nm, Supporting Information, Section 2.5, Figure SF5) pulled out of borosilicate capillaries and filled with either 10 μM fluorescein (FL) or a conjugate of dextran 70 kDa with fluorescein isothiocyanate (FITC-Dextran) in a physiological solution of 140 mM KCl, 20 mM HEPES 1% Sucrose (pH 7.5). Fluorescein contains two carboxylic acid groups, which dissociate in an aqueous solution and make it negatively charged. In contrast, FITC-Dextran has a low density of fluorescein substituents per glucose unit (1:250) in the polysaccharide chain. These substituents contribute minimal charge, and the overall dextran molecule is effectively neutral.

We integrated our probe into a SICM system with angular approach working in hopping mode^47,48^ (Supporting Information, Section S3.1, Figure SF6). Briefly, while the nanopipette approaches the cellular membrane, the ion-current flowing between the Ag/AgCl electrode inserted in the nanopipette and the Ag/AgCl electrode in the media with the cells is continuously recorded. This ion flow is generated by applying a constant voltage bias (200 mV) to the nanopipette electrode with respect to the reference electrode in the bath. When the nanopipette approaches the cellular membrane, a drop in the ion current to 99% of the baseline current is detected due to an increase in the access resistance at the nanopipette aperture (Supporting Information, Section 3.1, SF6). At this point, the nanopipette stops the approach at a distance from the cell equal to 2–3 radii (∼hundreds of nanometers). At this stage, we proceeded to move the nanopipette further until a ∼20% drop of the ion current is detected, and we delivered a train of electroporation pulses to temporarily permeabilize the plasma membrane and deliver the molecules within the mammalian cell (Figure 1E). A ∼ 20% drop ensures that the nanopipette is sufficiently close to the cell membrane to generate the necessary potential drop for effective electroporation. Our findings indicate that a minimum 20% decrease is required to achieve successful electroporation (Supporting Information, Section 3.2, Figure SF7). In the case of negatively charged fluorescein, we applied a train of 100 pulses with −10 V magnitude and 50 Hz frequency, while +10 V was used in the case of neutral FITC-Dextran. Both molecules were efficiently delivered in approximately ∼2 s of stimulation with minimal disruption of cellular morphology. Figure 1F shows an example of the delivery of fluorescein in a human breast cancer cell line (MCF7) and FITC-Dextran in mouse melanocytes (Melan-a). Fluorescein is a small molecule (332 Da) and rapidly diffuses through all cellular compartments, while FITC-Dextran has a molecular weight of 70 kDa and does not cross the nuclear pores, remaining in the cytosol, as observed in the figure. Furthermore, we quantified the amount of injected Dextran-FITC into MCF-7 cells in real time during the application of electroporation pulses (Figure 1G), and we found a linear relationship (R^2^ > 0.99) between the number of applied pulses and fluorescent intensity (i.e., number of delivered molecules), indicating that the number of injected molecules can be tightly controlled by the number of applied pulses (Supporting Information, section 3.3, Figure SF8). Our findings demonstrate that both charged and neutral molecules can be efficiently delivered into individual mammalian cells by leveraging the temporary permeabilization of the cellular membrane induced by electroporation pulses. This method enables rapid delivery (∼2 s), is noninvasive (does not require direct penetration of cell membrane), and provides precise control over the amount of cargo delivered, determined by the number of pulses applied.

### Spatially Controlled Delivery in the Nucleus or Cytoplasm

We challenged our system to achieve compartmental delivery specifically to the cytoplasm or nucleus of mammalian cells. We reasoned that by targeting the nuclear region and moving the nanopipette into close proximity with the cell, we could induce slight deformation of the plasma membrane against the nuclear envelope, enabling the applied electric potential to span both membranes. In this setup, the application of electroporation pulses would enable the temporary permeabilization of both the cytoplasmic membrane and nuclear envelope, delivering the molecules directly into the nucleus. To test our hypothesis, we used FITC-Dextran 70 kDa, which is large enough to prevent translocation through the nuclear pores, and its localization serves as an indicator of successful nuclear or cytoplasmic delivery. Figure 2A illustrates our setup in the case of cytoplasmic delivery. The nanopipette moves toward the plasma membrane in a cytoplasmic location which is far from the nucleus, and it stops upon reaching the SICM set point value (99% of baseline current) in the proximity of the plasma membrane. At this point, potential pulses with magnitude +10 mV and duration 50 ms are applied to visually monitor the ion current changes while the nanopipette was moved toward the cellular membrane with 500 nm steps using the SICM in manual mode. When the detected current reaches ∼ 85% of the initial value (*i*_*f*_ */i*_*i*_ = 0.85), the electroporation pulse train is applied (10 V, 3 ms, 50 Hz) and molecules are efficiently delivered into the cell cytoplasm. The set point for cytoplasmic delivery was obtained with a median indentation of 4 μm (Supporting Information, Section 3.4, Figure SF9). Figure 2B shows a typical ion current trace observed in the case of a pulse in the initial (blue) and final (orange) phases of the approach. Similarly, Figure 2C shows the setup used in the case of nuclear delivery. This time, the nanopipette is positioned on top of the nuclear region of the cell. It approaches the cellular membrane until the ion current reaches ∼50% of the initial value (*i*_*f*_ */i*_*i*_ = 0.50), ensuring the proximity of the plasma membrane to the nuclear envelope (Figure 2D). The set point value for nuclear delivery was obtained with a median indentation of 7.5 μm (Supporting Information, Section 3.4, Figure SF9). At this point, the same pulse train is applied (10 V, 3 ms, 50 Hz) to permeabilize both the cytoplasmic and nuclear membrane. We found that the *i*_*f*_ */i*_*i*_ parameter is crucial for determining whether the delivery will happen in the cytoplasm or nucleus. Figure 2E shows the distribution of the *i*_*f*_ */i*_*i*_ parameter in the case of cytoplasmic (blue) and nuclear (green) delivery. To achieve cytoplasmic delivery, we found that values of *i*_*f*_ */i*_*i*_ between ∼ 0.8 and 1 (median of 0.86) are sufficient to achieve efficient delivery. In the case of the nucleus, it is important to ensure proximity of the plasma membrane to the nuclear envelope by moving the nanopipette further down, and this results in *i*_*f*_ */i*_*i*_ between ∼ 0.4 and 0.6 (median = 0.47). Figure 2F–H shows images before and after delivery in the nucleus and cytoplasm of different cellular models. Figure 2F and Supporting Video 1 show a cytoplasmic delivery followed by a nuclear delivery in the same mouse melanocyte, demonstrating the precision of our system. Figure 2G,H shows the cytoplasmic and nuclear delivery into individual human embryonic stem cells, demonstrating the adaptability of our system to more complex cellular models. In particular, Figure 2H shows an example of nuclear delivery in a cell monolayer, demonstrating a high spatial resolution even in compact cellular environments with reduced accessibility. More images showing nuclear and cytoplasmic deliveries in different cell types are available in Supporting Information, Section 3.5, Figure SF10, and Supporting Videos 2–4. Our system enables the compartmental delivery of molecules with rapid execution, minimal cellular disruption, unparalleled precision in spatial localization, and near 100% reproducibility regardless of the cellular model.

**Figure 2:**
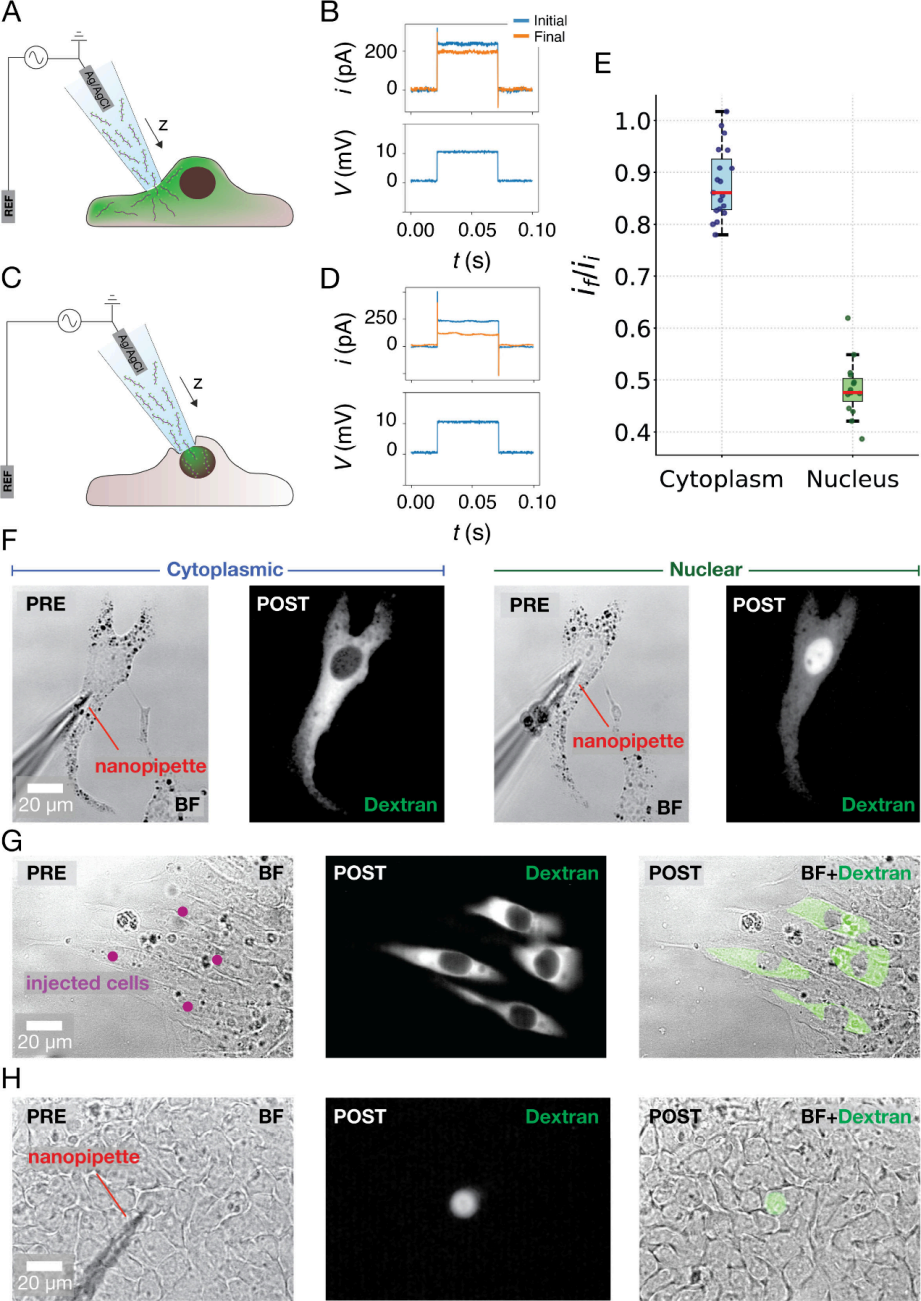
Spatially controlled delivery into the cytoplasm and/or nucleus of individual mammalian cells. (A) Illustration of the setup used for cytoplasmic delivery. The nanopipette moves toward the cell along the *z* axis until it reaches the SICM set point. At this point, voltage pulses are applied to the Ag/AgCl electrode to detect the ion current drop (∼15%) due to increased proximity to plasma membrane (B). For nuclear delivery (C), the same procedure is applied, but this time the current drop detected (D) equals to ∼ 50% the initial current, ensuring proximity of the plasma membrane to the nuclear envelope. (E) Boxplots showing the ratio between the ion current magnitude measured in the final (i_f_) and initial (i_i_) stage of the approach in the case of cytoplasmic (blue, *N* = 19) and nuclear (green, *N* = 15) delivery. (G and H) Optical and fluorescent micrograph obtained before and after the delivery of FITC-Dextran into the cytoplasm and nucleus of the same mouse melanocyte (F), in the cytoplasm of human embryonic stem cells (G), and into the nucleus of an individual human embryonic stem cell in a cell monolayer (H).

### Molecule Properties as Determinant of Pulse Shape Design

Molecular transport within a nanopipette is influenced by complex factors, which need to be considered alongside the properties of the molecule when designing a delivery experiment. The system introduced in this work can deliver molecules within mammalian cells, regardless of molecular charge. However, other properties can also impact transport. To explore this, we tested the system with different biomolecules including proteins and double-stranded DNA. Figure 3A presents our experimental question, which is to determine the optimal pulse shape for delivering molecules beyond dextran, such as α-synuclein (α-syn) monomers and plasmid DNA. α-Syn is a 140 residue intrinsically disordered protein whose aggregation into amyloid fibrils contribute to the formation of Lewy bodies, a hallmark of Parkinson’s disease.^49^ The C-terminus of the protein is highly acidic, with a net charge between −9 and −12 at physiological pH,^50^ conferring to the protein an isoelectric point of ∼4.7, which means that the protein carries negative charge at physiological pH. To enable fluorescent imaging of the monomers, we used an Alexa Fluor 488-labeled α-syn variant (α-syn–AF488).^51^ The fluorophore was site-specifically conjugated to the protein via maleimide–thiol chemistry at a Cys residue introduced at position 140, replacing the native Ala. While labeling introduces a minor charge modification, it is not expected to affect the overall behavior of the α-syn monomers. Given the strong negative charge, we reasoned that the protein could be delivered via electroporation by using a pulse train with negative polarity. To test whether α-syn can be effectively delivered by our system, we used a nanopipette filled with a 2 μM solution of α-syn–AF488 in PBS. These monomers were delivered into primary dorsal root ganglia (DRG) neurons isolated from rat, a clinically relevant cellular model.

**Figure 3:**
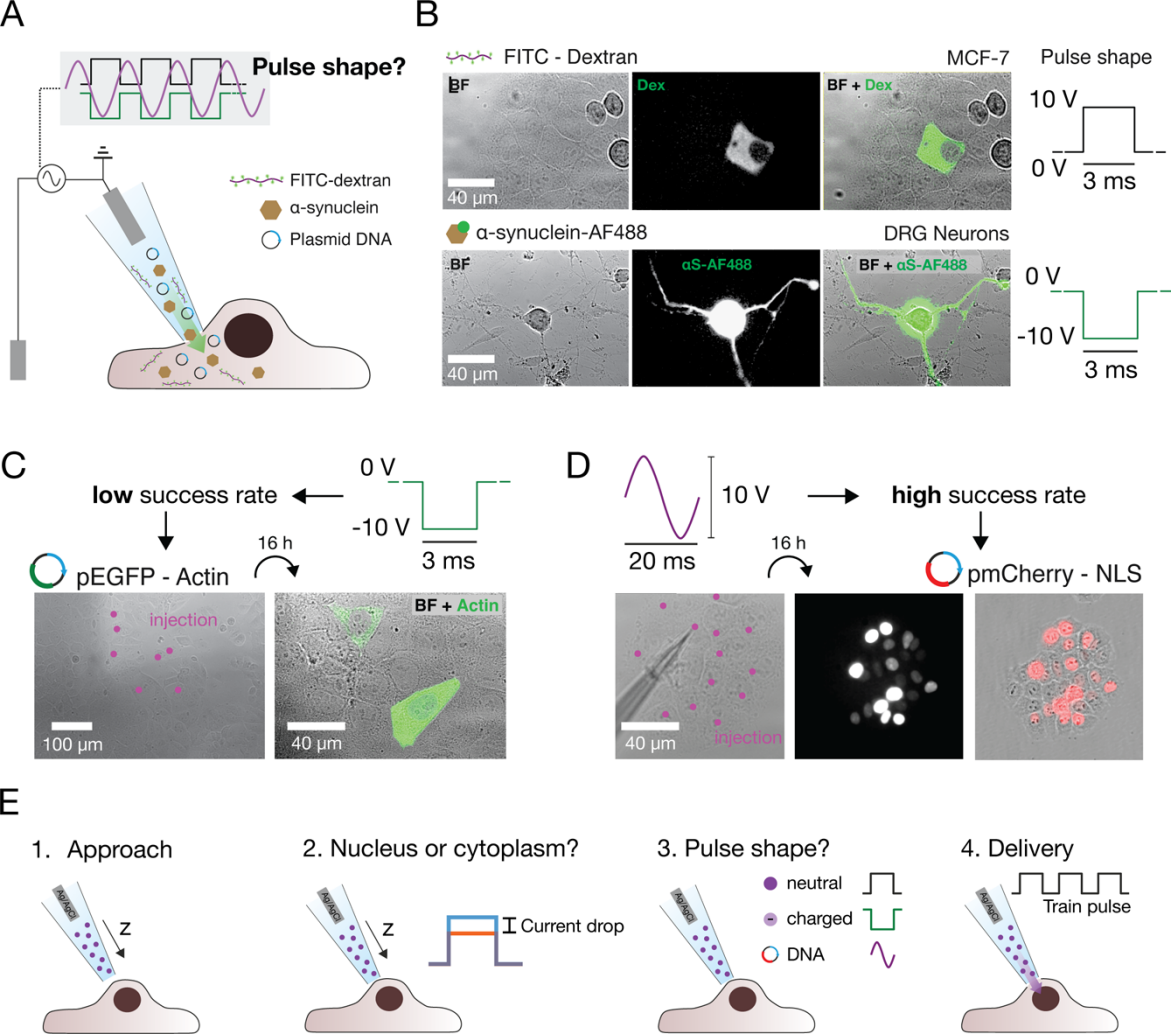
Molecule properties are determinant of pulse shape design. (A) Illustration of the system employed in this work to deliver biomolecules with different properties. (B) Optical and fluorescent micrographs before and after delivery of neutrally charged FITC-Dextran (positive pulse) into MCF-7 and negatively charged α-syn–AF488 (negative pulse) into rat primary DRG neurons. (C) Optical and fluorescent micrograph before and 16 h post transfection showing the low success rate obtained in the case of pEGFP-Actin delivery into MCF-7 cells using a square pulse with negative polarity. (D) Optical and fluorescent micrograph before and 16 h post transfection showing the high success rate obtained in the case of pmCherry-NLS delivery into MCF-7 using a sine wave as pulse shape. (E) Illustration of future development of the system, where the nanopipette automatically identifies and approaches the cell cytoplasm or nuclear region (1), moves toward the cell/nuclear membrane until a preset current drop is detected (2), chooses the pulse shape depending on the molecule to inject (3), and delivers the preset train pulse (4).

The nanopipette was positioned above the cell soma and directed toward the cellular membrane. The application of a pulse train of 50 square pulses (−10 V, 3 ms, 50 Hz) resulted in the delivery of monomers into the cell. Figure 3B shows the optical and fluorescent micrographs before and after the delivery of α-syn in a rat DRG neuron compared to the delivery of FITC-dextran into a human breast cancer cell (MCF-7) using positive polarity. Additional images showing α-syn are available in Supporting Information, Section S4.1, Figure SF11. The amount of delivered monomers was sufficient to saturate the fluorescent signal in the cell soma. We chose this approach to ensure that the monomers would not only enter the soma but also travel to the dendrites, allowing us to confirm that the monomers were delivered throughout the entire cell including its distal regions. This confirms that knowing the charge properties of the molecule is a key aspect of defining the shape of the pulse.

Next, we applied the same approach to deliver double-stranded DNA, which carries a negative charge at physiological pH due to its phosphate backbone. We attempted to deliver a 5.8 kbp plasmid encoding cytoplasmic actin tagged with the EGFP reporter (pEGFP-Actin) and a 4.7 kbp plasmid encoding the fluorescent nuclear reporter pmCherry-NLS using a train pulse of 100 negative square pulses (−10 V, 3 ms, 50 Hz). However, successful delivery and transfection were limited to only the first 1–2 electroporated cells out of more than 7 attempts, as shown in Figure 3C for pEGFP-Actin. Unsuccessful delivery was likely due to DNA trapping at the nanopipette aperture, as previously observed.^52^ We reasoned that applying a symmetric sine wave would enable efficient plasmid DNA delivery, as DNA is trapped during the negative half cycle and released during the positive half cycle.^52^ Applying a sine wave (±5 V, 20 ms, 50 Hz) resulted in 100% successful delivery, with all cells transfected and viable 16 h post transfection, as shown in Figure 3D for pmCherry-NLS. Efficient transfection was confirmed by the nuclear pmCherry-NLS signal. The increase in the number of pmCherry-NLS positive cells 16 h post-transfection (n = 22), compared to the number of initially injected cells (n = 13), suggests that most cells underwent division during this period, indicating that the delivery was nondisruptive and did not interfere with cell cycle progression.

## Discussion

The work presented here highlights several key aspects of voltage-induced flow generation in nanopipettes and its implications for molecular transport. Specifically, we observed that the application of voltage within a nanopipette generates bulk flow of liquid that can influence the movement of molecules in and out of the nanopipette. This phenomenon is also influenced by the nonlinear properties of glass nanopipettes, particularly the formation of the electric double layer^42^ at the negatively charged glass wall, which can induce electroosmotic flow.

The capability of this system to generate flow in response to voltage pulses makes it applicable to nanopipette-based single-cell electroporation. We successfully integrated our nanopipette with a SICM for precise positioning, and we used it to electroporate both neutral molecules (via voltage-driven liquid flow) and charged molecules (via electrophoretic flow) into various mammalian cellular models. While our study focused on neutral and negatively charged molecules, additional considerations—such as surface modifications of the nanopipette—may be required to enable efficient delivery of positively charged cargo due to potential electrostatic interactions with the negatively charged glass surface. The capability of SICM for real-time monitoring enabled us to track the ion current during electroporation, facilitating precise and compartment-specific delivery into the nucleus or cytoplasm with nearly 100% reproducibility without physically penetrating the cell membrane. While the extent of nanopipette indentation may vary with cell stiffness, our consistent electroporation results across different cell types with markedly different mechanical properties (e.g., human embryonic stem cells vs breast cancer cells) suggest that the delivery is not strongly influenced by membrane stiffness once the target set point is reached.

Our findings underscore the importance of considering the physicochemical properties of molecules when designing electroporation pulse shapes. As a proof-of-concept, we successfully delivered α-syn, the key protein in Parkinson’s disease, into DRG neurons, as well as plasmid DNA, which exhibited unconventional transport properties within the nanopipette.

Future work will focus on the development of a fully automated SICM-based system capable of identifying the cell, approaching the cellular membrane, detecting the ion current drop specific for nuclear/cytoplasmic injection, and selecting and delivering the optimal pulse shape, as illustrated in Figure 3E, with significant potential in both fundamental biological research and therapeutic applications. While the angular approach offers improved visualization beneficial for prototyping, a vertical approach could be used without compromising performance, although some parameter optimization may be required. Future work will also explore delivery to other organelles, although effective delivery relies on the proximity of the organelle to the plasma membrane to ensure sufficient voltage drop across both membrane without requiring penetration. We also expect that the voltage-induced liquid flow introduced will enable new approaches for SICM-based stiffness mapping.

Finally, our findings highlight the need to tailor electroporation parameters to each molecule as the standard one-size-fits-all strategy is ineffective.

## Supplementary Material





## Data Availability

All data supporting the findings of this study are publicly available on Zenodo (DOI: 10.5281/zenodo.15850920).

## Notes

Video 1, subsequent cytoplasmic and nuclear delivery in the same mouse melanocyte (4x speed); Video 2, subsequent cytoplasmic delivery into MCF-7 cells with the same nanopipette (8x speed); Video 3, nuclear delivery into an MCF-7 cell (4x speed); Video 4, cytoplasmic delivery into a mouse melanocyte (4x speed) (ZIP)
Materials and methods, characterization of electrochemical nanoprobe for voltage-induced liquid flow experiment, increase in faradaic current in response to applied pressure, characterization of voltage-induced flow in case of neutral mediator, characterization of voltage-induced flow in case of charged mediator, nanopipette aperture size estimation based on electrical resistance, SICM ion current traces, minimum current drop for efficient electroporation, pulse-dependent delivery of molecules, probe indentation during cytoplasmic and nuclear delivery, nuclear delivery in different cell lines, delivery of fluorescently tagged α-synuclein monomers into DRG neurons (PDF)

## ⬢

Institute of Chemical Biology, Molecular Sciences Research Hub, Imperial College London, London W12 0BZ, U.K
