# Supplementary material for: Delivery of Biomolecules into Individual Cells and Subcellular Compartments by Localized Electroporation via Nanopipette
Source: ACS Nanosci Au. 2025 Aug 1;5(5):353–61. doi: 10.1021/acsnanoscienceau.5c00053 (PMC12531870; doi:10.1021/acsnanoscienceau.5c00053)
Supplement: Supplementary file 2 [file ng5c00053_si_002.pdf]

Supporting Information:

Delivery of biomolecules into individual cells and sub-cellular compartments by localized electroporation via nanopipette.

Fabio Marcuccio[1,2,*], *Philip S. Goff*[3], *Devkee M. Vadukul*[4], *Fawaz Raja*[4], *Yilin Li*[4], *Ren Ren*[1,4,7], *Debjani Saha*[5], *Luca Magnani*[5], *Francesco A. Aprile*[4], *Uma Anand*[1], *Elena V. Sviderskaya*[3], *Joshua B. Edel*[4], *Aleksandar P. Ivanov*[4], *Petr V. Gorelkin*[6], *Yuri Korchev*[1,7] *and Andrew Shevchuk*[1,*].

[1] Faculty of Medicine, Imperial College London, Hammersmith Campus, Du Cane Road, London, W12 0NN, UK

[2] Genomics Research Centre, Human Technopole, Viale Rita Levi-Montalcini 1, Milan, 20157, Italy

[3] School of Health and Medical Sciences, City St George's, University of London, Cranmer Terrace, London, SW17 0RE, UK

[4] Department of Chemistry, Imperial College London, Molecular Sciences Research Hub, White City Campus, 82 Wood Lane, London, W12 0BZ, UK

[5] The Breast Cancer Now Toby Robins Research Centre, The Institute of Cancer Research, 123 Old Brompton Road, London, SW7 3RP, UK

[6] ICAPPIC Limited, London, NW10 6TD, UK

[7] WPI Nano Life Science Institute (WPI-NanoLSI), Kanazawa University, Kanazawa 920-1192, Japan

* Corresponding authors. Correspondence to a.shevchuk@imperial.ac.uk and fabio.marcuccio@fht.org

Contents

S1: Materials and Methods 3
S1.1 Nanopipette fabrication 3
S1.2 Voltage-induced liquid flow experiment 3
S1.3 Single-cell electroporation 4
S1.4 Single-cell electroporation and simultaneous imaging of delivered cargo 5
S1.5 SICM setup 5
S1.6 α-synuclein monomers tagged with alexafluor 488 6
S2: Voltage-induced liquid flow within a nanopipette 7
S2.1 Characterization of electrochemical nanoprobe 7
S2.2 Increase in faradaic current in response to applied pressure 8
S2.3 Characterization of voltage-induced flow in case of neutral mediator 9
S2.4 Characterization of voltage-induced flow in case of charged mediator 10
S2.5 Nanopipette aperture size estimation based on electrical resistance 11
S3: Sub-cellular delivery via electroporation 12
S3.1 SICM traces 12
S3.2 Minimum current drop for efficient electroporation 13
S3.3 Pulse-dependent delivery of molecules 14
S3.4 Probe indentation during cytoplasmic and nuclear delivery 15
S3.5 Nuclear delivery in different cell lines 15
S4: Molecule properties as determinant of pulse shape 17
S4.1 Delivery of fluorescently tagged α-synuclein monomers into DRG neurons 17
S5: References 18

S1: Materials and Methods

S1.1 Nanopipette fabrication

Double-barrel nanopipettes were pulled from quartz capillaries (OD 1.2 mm, ID 0.9 mm, QT120-90-7.5, World Precision Instrument) using a CO_2 Laser Puller (P2000, Sutter Instrument). A single-line program was used with the following parameters: HEAT 700, FILAMENT 3, VELOCITY 45, DELAY 130, PULL 93. A previously published protocol developed by our group was used to deposit pyrolytic carbon in one barrel[1]. Briefly, the barrel was filled with propane/butane and heated under an inert atmosphere (Argon) with a butane flame to decompose the carbon gas and deposit pyrolytic carbon inside the nanopipette. Single-barrel nanopipettes for the electroporation experiments were pulled out of borosilicate capillaries (OD 1.0 mm, ID 0.5 mm, B-100-50-10, World Precision Instrument) using the same laser puller using a pulling program with the following parameters: Line 1: HEAT 280, FILAMENT 3, VELOCITY 20, DELAY 150, PULL 0 /Line 2: HEAT 300, FILAMENT 4, VELOCITY 15, DELAY 120, PULL 100. It should be noted that pulling parameters are instrument specific.

S1.2 Voltage-induced liquid flow experiment

A silver wire was inserted into the carbon barrel of the double-barrel nanopipette to establish contact with the carbon electrode. An Ag/AgCl electrode was inserted in the open barrel, and the barrel was filled with an electrolyte solution containing/not containing the redox mediator for the experiment/control. In the case of a neutral mediator, a 1 mM solution of ferrocenemethanol ($FcCH_2OH$) in 100 mM KCl was used, while a solution of 1 mM hexa ammineruthenium(III) chloride ($[Ru(NH_3)_6]Cl_3$) in 100 mM KCl was used for the charged mediator. In both cases, the control solution was 100 mM KCl. The double-barrel nanopipette was immersed in an electrolyte bath filled with 1 mM $FcCH_2OH$/$[Ru(NH_3)_6]Cl_3$ in 100 mM KCl and a Ag/AgCl electrode was immersed in the bath and used as reference. The carbon electrode was kept at constant 0.3 V while different potential programs (ramp, steps, pulse) were applied to the Ag/AgCl electrode in the open barrel. Electric

potential was applied to the Ag/AgCl reference electrode in the nanopipette using the analog out channels of an Axon Digidata 1550B (Molecular Devices) analog-to-digital converter. The electric potential programs were generated using Clampex (Molecular Devices). The back end of the double-barrel nanopipette was connected to a home-built pressure pump equipped with a controller via rubber tubbing using a 3-way valve for the application of pressure.

S1.3 Single-cell electroporation

A single-barrel borosilicate nanopipette was filled with a solution containing 140 mM KCl, 20 mM HEPES, and 1% sucrose (pH 7.5), along with the molecule of interest, including 10 μM FITC-Dextran 70 kDa (Sigma-Aldrich 46945), 1 μM fluorescein (Sigma-Aldrich F6377), 2 μM α-synuclein conjugated to AlexaFluor 488, 10 nM plasmid DNA (pmCherry-NLS, Addgene #39319, pEGFP-actin, Novoprolabs V001212). The nanopipette was integrated into a custom-built angular approach scanning ion-conductance microscope (SICM) operating in hopping mode[2,3]. The nanopipette was mounted at an angle of ~30° relative to the petri dish containing the cells. Mammalian cells were seeded in a 35 mm dishes in their culture medium. An Ag/AgCl electrode was placed in the bath solution containing the cells, while another Ag/AgCl electrode was inserted into the nanopipette. The electric potential was applied to the bath electrode with respect to the pipette electrode. This configuration allowed for the application of electric potentials greater than 1 V using standard patch-clamp amplifiers (Axopatch 200B, Molecular Devices) and digitizers (Digidata 1440, Molecular Devices), which are typically limited to a voltage range of -1 V to 1 V. For nanopipette positioning, a micromanipulator (Scientifica) was used to manually align it above the target region on the cell. The SICM feedback control monitored the ion current until a preset feedback threshold (99% of baseline current) was reached, corresponding to a nanopipette-cell distance of 2-3 times the nanopipette aperture radius (**Section S3.1, Figure SF6**). Following detection of this threshold, continuous electric pulses (10 mV amplitude, 50 ms duration) were applied to the nanopipette electrode while the pipette was moved in 500 nm steps towards the cellular membrane using manual SICM mode. The pulses enabled clearer visualization of the ion current drop after each step. This process continued until a 15%/50% drop in ion current was detected, corresponding to targeting the cytoplasm/nucleus,

respectively. At this point, an electroporation pulse train was delivered via the analog output channel of the digitizer (Digidata 1440, Molecular Devices). The electroporation parameters were tuned to the molecule being delivered: neutral molecules, 100 square pulses, 10 V, 3 ms duration, 50 Hz; negatively charged molecules, 100 square pulses, -10 V, 3 ms duration, 50 Hz; plasmid DNA, 100 sine pulses, 10 V peak-to-peak (-5 V, +5 V), 20 ms duration, 50 Hz). For clarity, the voltage polarity is described throughout the article as if it were applied to the nanopipette electrode relative to the reference electrode. However, in practice, the potential was applied to the reference electrode relative to the nanopipette electrode. The entire procedure was imaged using an upright epifluorescent microscope (Olympus). All experiments were carried out at room temperature and lasted less than 30 minutes.

S1.4 Single-cell electroporation and simultaneous imaging of delivered cargo

To quantify the amount of delivered cargo and its correlation with the number of electroporation pulses (**Figure 1G**), we employed a vertical approach SICM operating in hopping mode[41] to inject FITC-Dextran 70 kDa into the cytoplasm of individual MCF-7 cells. The nanopipette was positioned over a cytoplasmic region of the cell, and electroporation pulses were applied following the cytoplasmic injection protocol using the angular approach described in the manuscript. During pulse application, fluorescent intensity was measured using a photomultiplier connected to the input port of the digitizer (Multiclamp 1550B, Molecular Devices). Additional details can be found in **Section S3.3, Figure SF8**.

S1.5 SICM setup

Experiments were performed using previously published Angular Approach Scanning Ion Conductance Microscope[36] installed on Olympus BX-51WI upright optical microscope (Japan) and equipped with ICAPPIC Universal Controller (ICAPPIC Limited, UK). Ion current was registered using Axopatch 200B amplifier and recorded using Digidata 1440 (Molecular Devices, USA). Brightfield and fluorescence images were recorded using Watec WAT-902H Ultimate camera (Japan).

S1.6 α-synuclein monomers tagged with alexafluor 488

α-Syn carrying the A140C mutation (α-syn A140C) was purified using a previously reported protocol[4]. Briefly, the protein was overexpressed in E. coli BL21(DE3) overnight with 1 mM IPTG, followed by cell lysis, streptomycin sulfate precipitation of nucleic acids, and ammonium sulfate precipitation of proteins. α-Syn was further purified by ion-exchange chromatography on a HiPrep Q HP 16/10 column (Cytiva) using a 0–1 M NaCl linear gradient, followed by size-exclusion chromatography on a HiLoad 16/600 Superdex 75 pg column (GE Healthcare) in standard PBS. All steps were performed in the presence of 1 mM DTT.

Labelled α-syn monomers were obtained by conjugation of A140C α-syn with Alexa Flour 488 C5-maleimide (Thermo Fisher Scientific). A140C α-syn was buffer-exchanged using Zeba Spin Desalting Columns (7K molecular weight cutoff) (Thermo Fisher Scientific) to remove the DTT from the storage solution. The reaction was carried out according to the manufacturer's instructions.

S2: Voltage-induced liquid flow within a nanopipette

S2.1 Characterization of electrochemical nanoprobe

The open barrel of the double barrel nanopipette was filled with a solution of 0.1 M KCl. The nanopipette was immersed in a bath containing a solution of 1 mM ferrocenemethanol ($FcCH_2OH$) in 0.1 M KCl. A voltage ramp (-400 mV, 400 mV) was applied to the Ag/AgCl electrode in the open barrel with respect to the Ag/AgCl electrode in the bath electrode. A voltage ramp from 0 to -400 mV, then to 400 mV, and back to 0 mV, with scan rate 130 mV/s was applied to the carbon electrode. The current response of the open barrel and the carbon electrode are shown in **Figure SF1**.

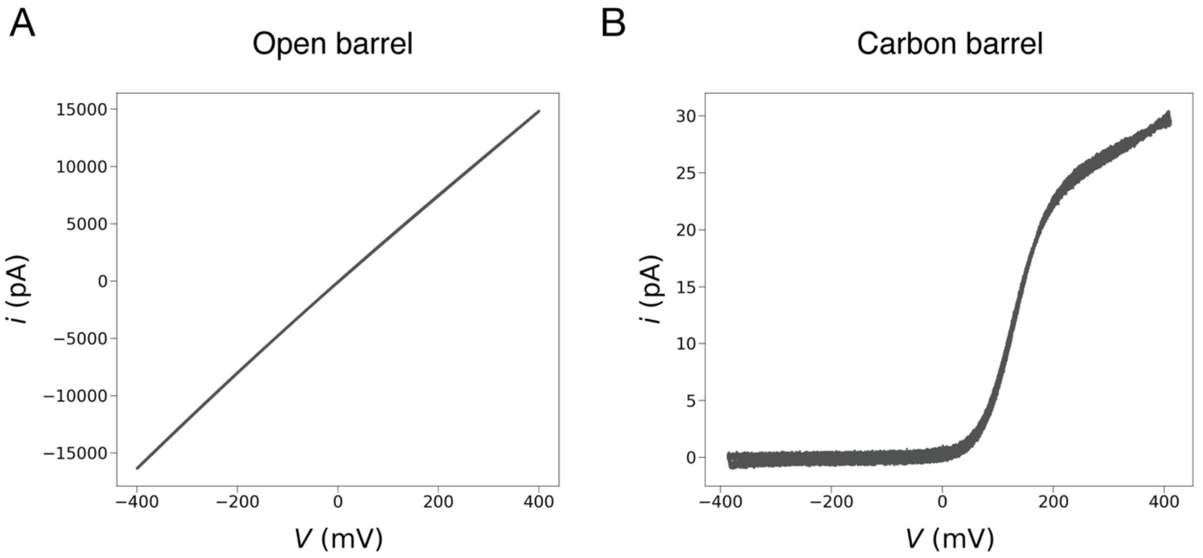

***Figure SF1**: Characterization of the double barrel electrochemical nanoprobe. Current response to a voltage ramp applied to the open (A) and carbon (B) barrel.*

S2.2 Increase in faradaic current in response to applied pressure

A pressure was created inside the nanopipette by connecting the back end of the nanopipette to a pump using rubber tubing and the Faradaic current measured at the carbon electrode was recorded under no voltage applied at the Ag/AgCl electrode in the open barrel. **Figure SF2** shows the interpolation of the pressure-current response using a linear curve.

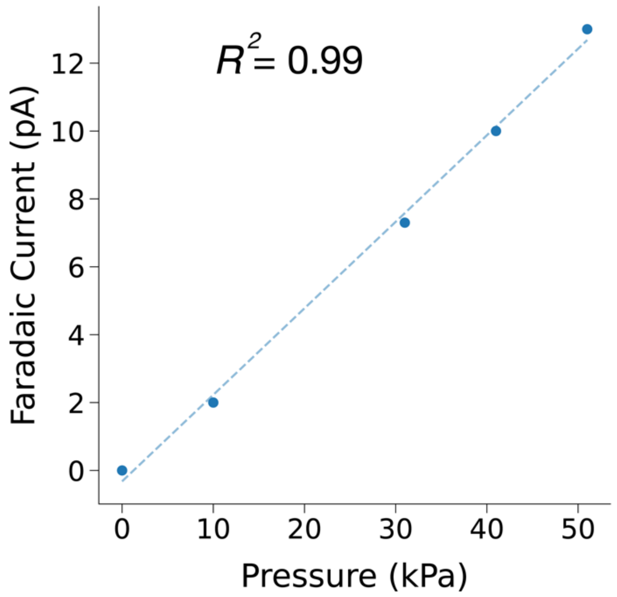

Figure SF2: *Faradaic current detected at the carbon electrode upon application of external pressure.*

S2.3 Characterization of voltage-induced flow in case of neutral mediator

The open barrel of the double-barrel nanopipette was loaded with a solution of 1 mM ferrocenemethanol (FcMeOH) in 0.1 M KCl **or** 0.1 M KCl (control) and was immersed in 0.1 M KCl bath. The electric potential at the carbon electrode with respect to the bath electrode was maintained at constant +300 mV (V_C), while a potential ramp/steps were applied to the Ag/AgCl electrode in the open barrel with respect to the same bath electrode. The linear current recorded at the electrode in the open barrel (i_{open}) is due to the ion current flowing between the Ag/AgCl in the barrel and the one in the bath, while the detection of a Faradaic current at the carbon electrode (i_C) indicates an outflow of mediator molecules, suggesting the presence of a voltage-induced liquid flow which carries the neutral mediator out of the barrel. **Figure SF3** shows the current response recorded at the Ag/AgCl electrode in the open barrel and the faradaic current at the carbon electrode in case of a ramp (A) or steps (B) of voltage applied to the Ag/AgCl electrode in the open barrel where the open barrel was filled with either 1 mM FcMeOH in 0.1 M KCl (green) or 0.1 M KCl (grey) as control. The bath solution was in both cases 0.1 M KCl.

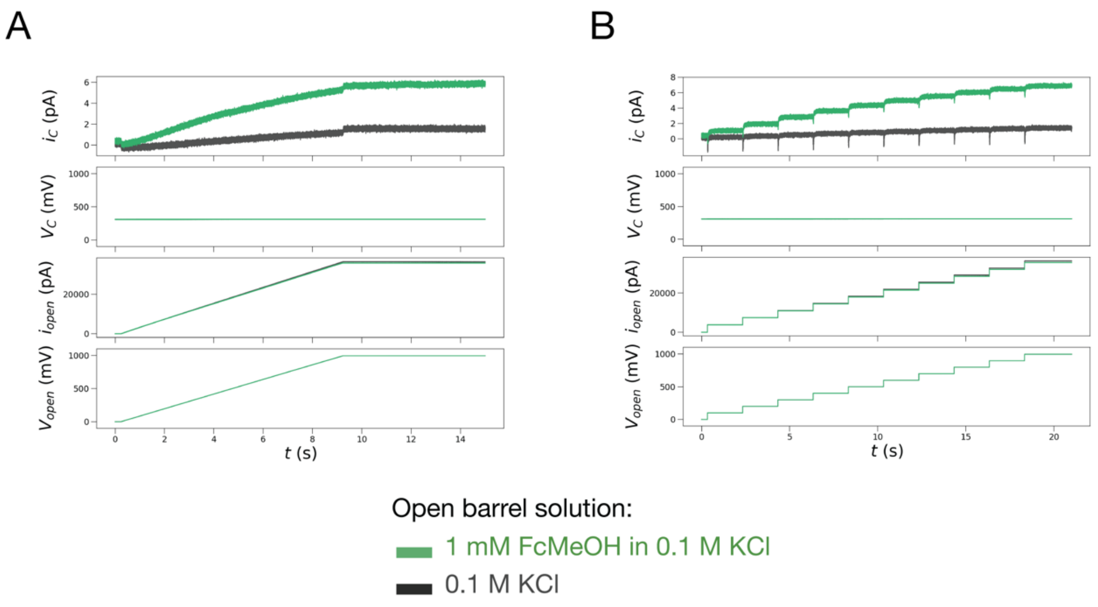

Figure SF3: *Characterization of voltage-induced flow in case of neutral mediator (FcMeOH). The open barrel of a double barrel nanopipette was filled with either 1 mM FcMeOH in 0.1 M KCl (green) or 0.1 M KCl (gray). The nanopipette was immersed in a 0.1 M KCl bath. The carbon barrel was held at a constant potential of 300 mV with respect to the Ag/AgCl electrode in the bath while a voltage ramp (A) or steps (B) were applied to the Ag/AgCl electrode in the open barrel with respect to the bath electrode.*

S2.4 Characterization of voltage-induced flow in case of charged mediator

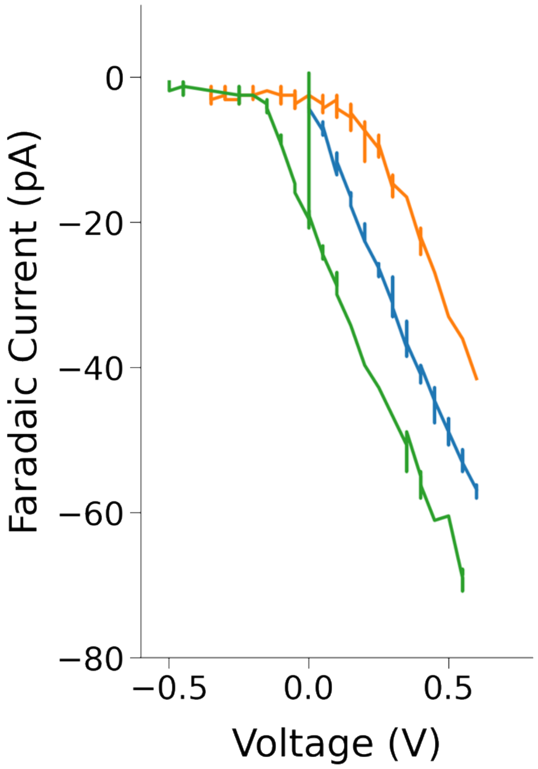

Figure SF4: *Faradaic current measured at the carbon electrode held at +300 mV under different pressure and voltage conditions in the case of charged mediator ([Ru(NH3)6]Cl3).*

S2.5 Nanopipette aperture size estimation based on electrical resistance

The nanopipette aperture dimensions can be estimated analytically from the measured electrical resistance by approximating the nanopipette geometry as a truncated hollow cone[5]. **Figure SF5** shows the estimation of the aperture radius for various resistances, assuming a glass nanopipette pulled out of capillaries with 1.0 mm outer diameter and 0.5 mm inner diameter, an inner half-cone angle of 3.5° and an electrolyte conductivity of 1.3 S/m (conductivity of 100 mM KCl).

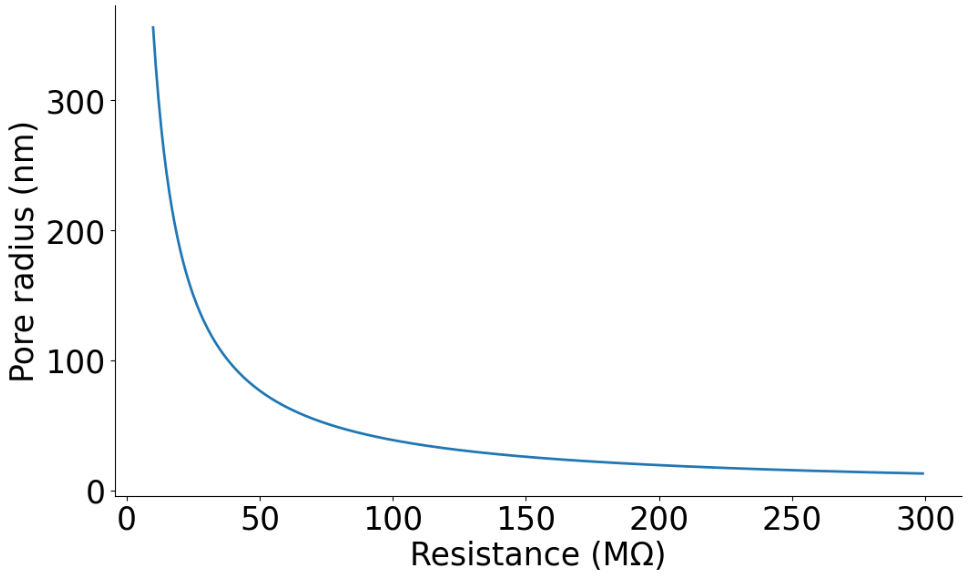

Figure SF5: *Estimation of nanopipette aperture radius based on measured electrical resistance.*

S3: Sub-cellular delivery via electroporation

S3.1 SICM traces

The SICM operating in hopping mode enables automatic approach of the cellular membrane. **Figure SF6** shows the typical current traces for the ion current *i*, the voltage applied *V*, and the *z-piezo* parameter recorded using two different nanopipettes while they approach the cellular membrane. A constant voltage bias $V = 200$ mV is applied to the inner electrode of the nanopipette relative to the bath electrode while the nanopipette moves toward the cellular membrane. As the nanopipette approaches the membrane, the ion current *i* decreases due to an increase in the access resistance at the nanopipette aperture, caused by the membrane hindering ion flow. The SICM stops the nanopipette movement when the ion current *i* drops to 99% (~4100 pA for nanopipette 1, ~5800 pA for nanopipette 2) of its baseline value (~4140 pA for nanopipette 1, ~5860 pA for nanopipette 2). At this point, the piezoelectric motor retracts the nanopipette by a distance equal to the hopping height parameter set to 1 μm. This approach-retract cycle is then repeated until a new command is issued, resulting in the characteristic "sawtooth" pattern observed in **Figure SF6**. Note that a *z-piezo* indicates the position of the SICM piezoelectric motor (not the distance from the cell) and a higher value for *z-piezo* means that the nanopipette is closer to the cellular membrane.

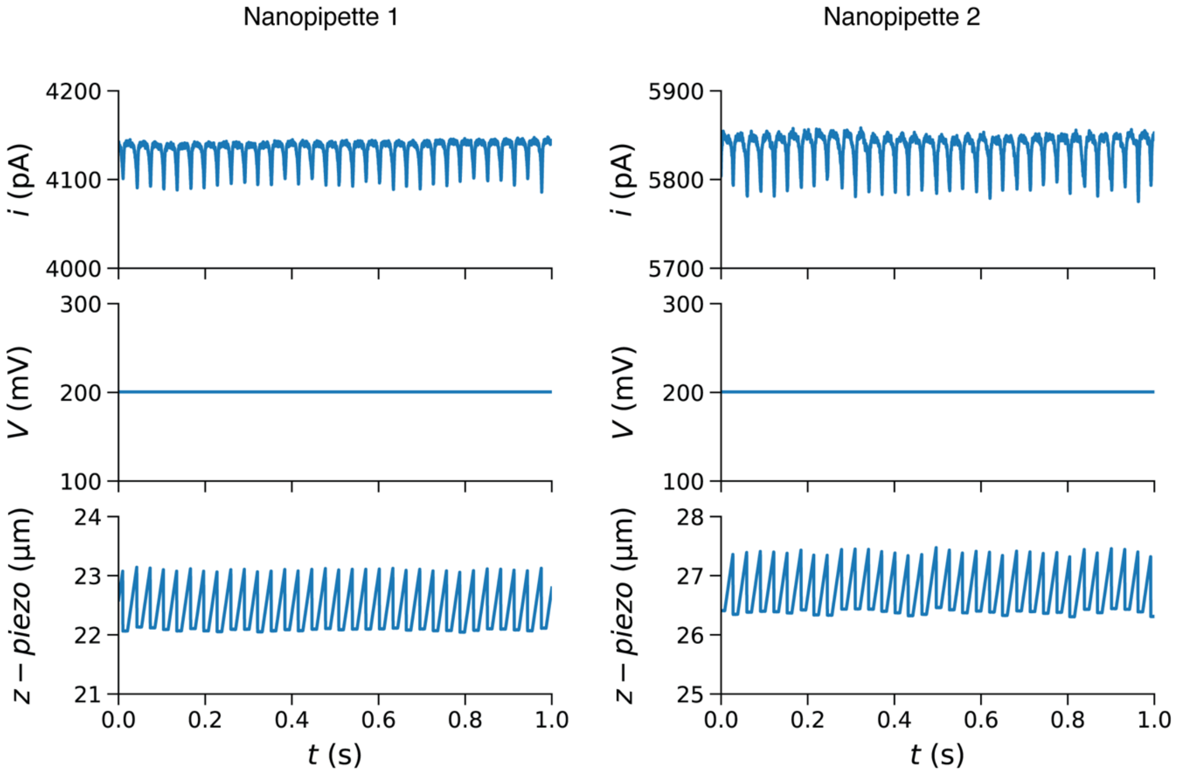

Figure SF6: *SICM traces for ion current (i), voltage applied (V) and z motion (z-piezo) recorded during the approach of the cellular membrane using two different nanopipettes. The SICM works in hopping mode with hopping height set to 1 μm and setpoint current equal to 1%.*

S3.2 Minimum current drop for efficient electroporation

To determine the optimal current drop for efficient cytoplasmic electroporation and cargo delivery, we used the same nanopipette filled with 10 μM FITC-Dextran 70 kDa in a 140 mM KCl, 20 mM HEPES, 1% Sucrose while varying the detected current drop during the cytoplasmic approach across different experiments. Our results showed that a minimum 20% current drop is required for effective cytoplasmic delivery, while drops greater than 40% result in nuclear delivery. **Figure SF7** shows the optical and fluorescent micrographs of electroporated HEK cells alongside the corresponding current drop detected during nanopipette approach.

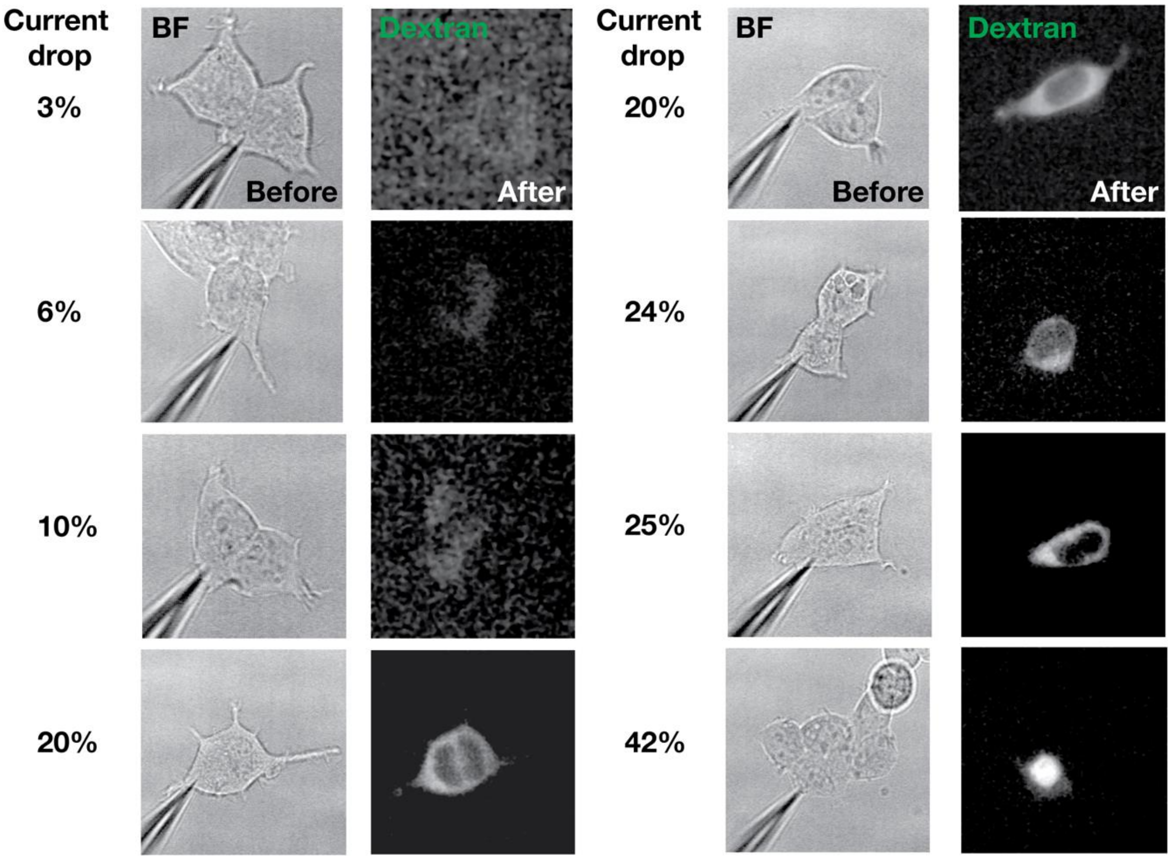

Figure SF7: *Minimum current drop for efficient electroporation. For current drops greater than 40%, nuclear electroporation is achieved. The experiment was performed in HEK293T cells. 100 electroporation pulses were applied for all cells.*

S3.3 Pulse-dependent delivery of molecules

The fluorescence intensity curves, recorded during the delivery of FITC-Dextran 70 kDa into individual MCF-7 cells (**Fig. SF8A**), were measured using a photomultiplier attached to an inverted microscope. The signal was detected within a cytoplasmic region selected using an optical slit. A linear regression analysis of the mean curve from 10 replicates confirmed a linear relationship (R^2=0.99) between the number of pulses and fluorescence intensity (**Fig. SF8B**), indicating proportional cargo delivery.

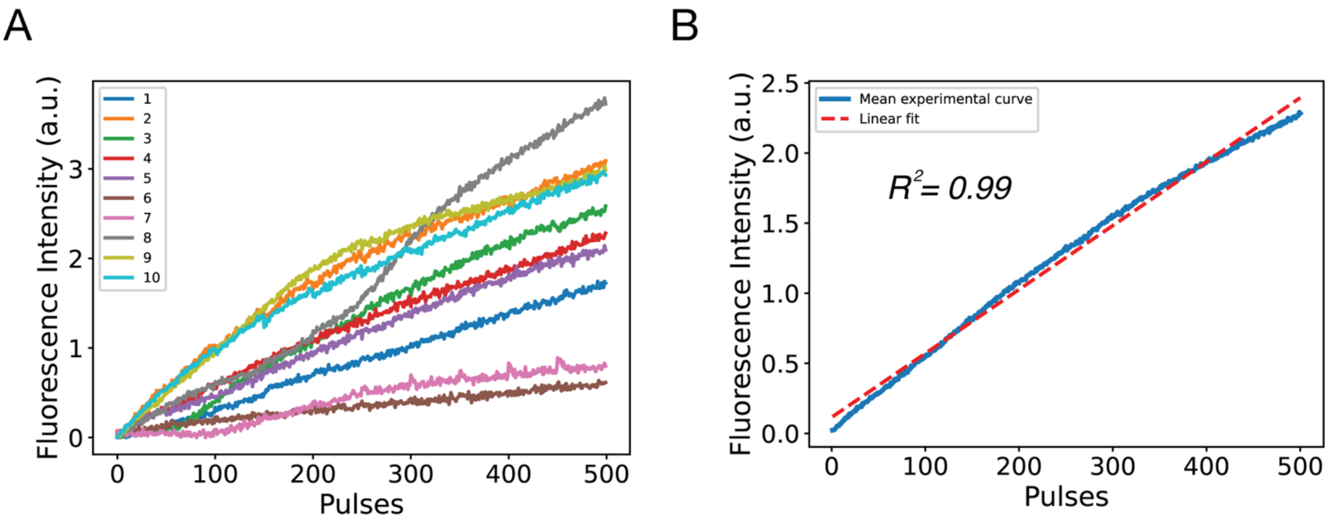

Figure SF8: *Pulse-dependent delivery of molecules. (A) Curves obtained from the delivery into 10 different individual cells. (B) Mean curve obtained from 10 replicates and linear fit obtained from linear regression.*

S3.4 Probe indentation during cytoplasmic and nuclear delivery

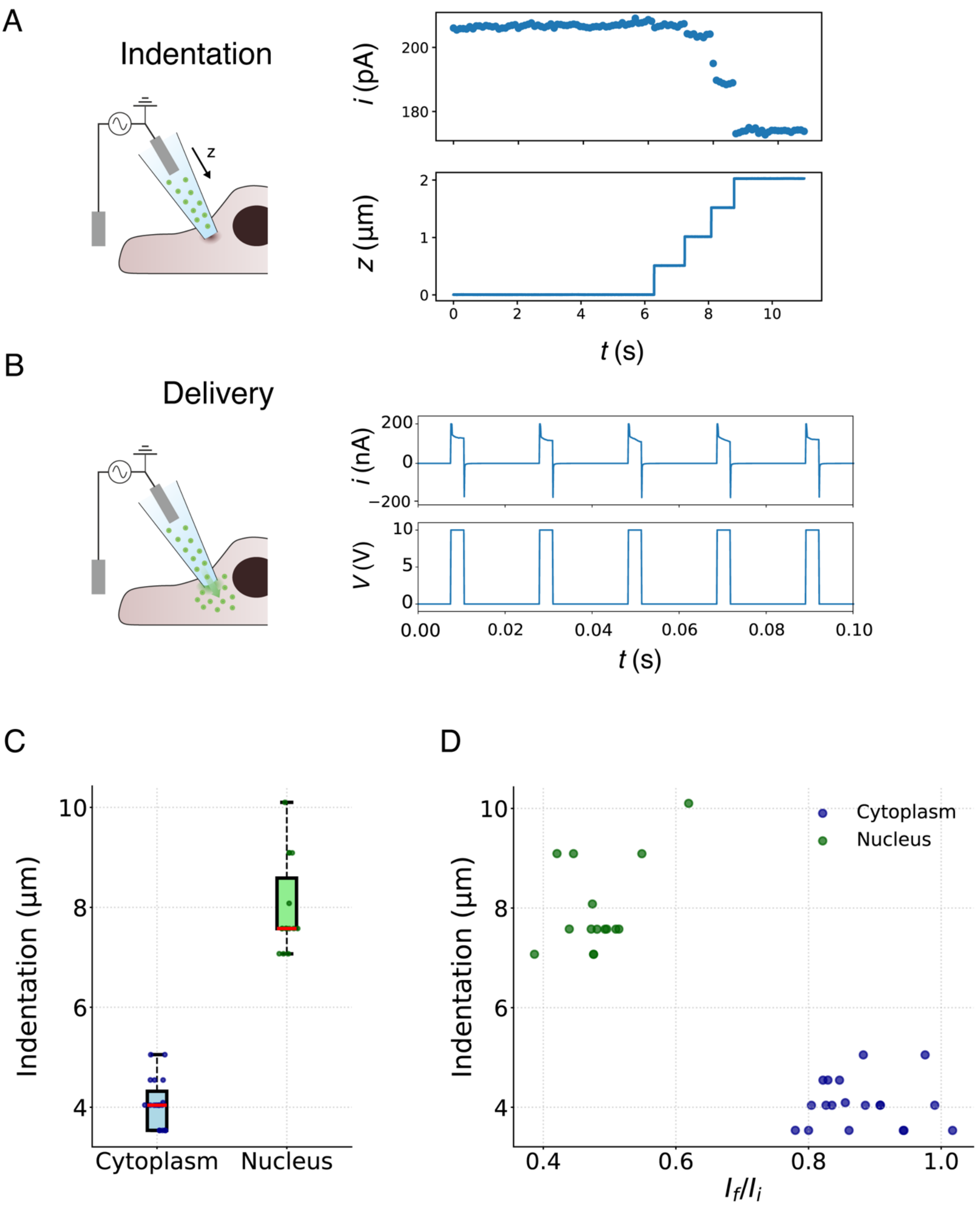

***Figure SF9**: Probe indentation during cytoplasmic and nuclear delivery. (A) Nanopipette approach toward the cellular membrane in 500 nm steps, with corresponding ion current amplitudes averaged over each individual 10 mV pulse. (B) Delivery of electroporation pulses (10 V, 3 ms, 50 Hz) and corresponding ion current magnitude generated. (D) Total indentation, calculated as the difference between the nanopipette position at the 1% setpoint and its final position during cytoplasmic and nuclear delivery. Note that indentation is also influenced by the approach angle, which was maintained at approximately 30°. (D) Scatter plot showing total indentation versus ion current magnitude ratio for both cytoplasmic and nuclear delivery.*

S3.5 Nuclear delivery in different cell lines

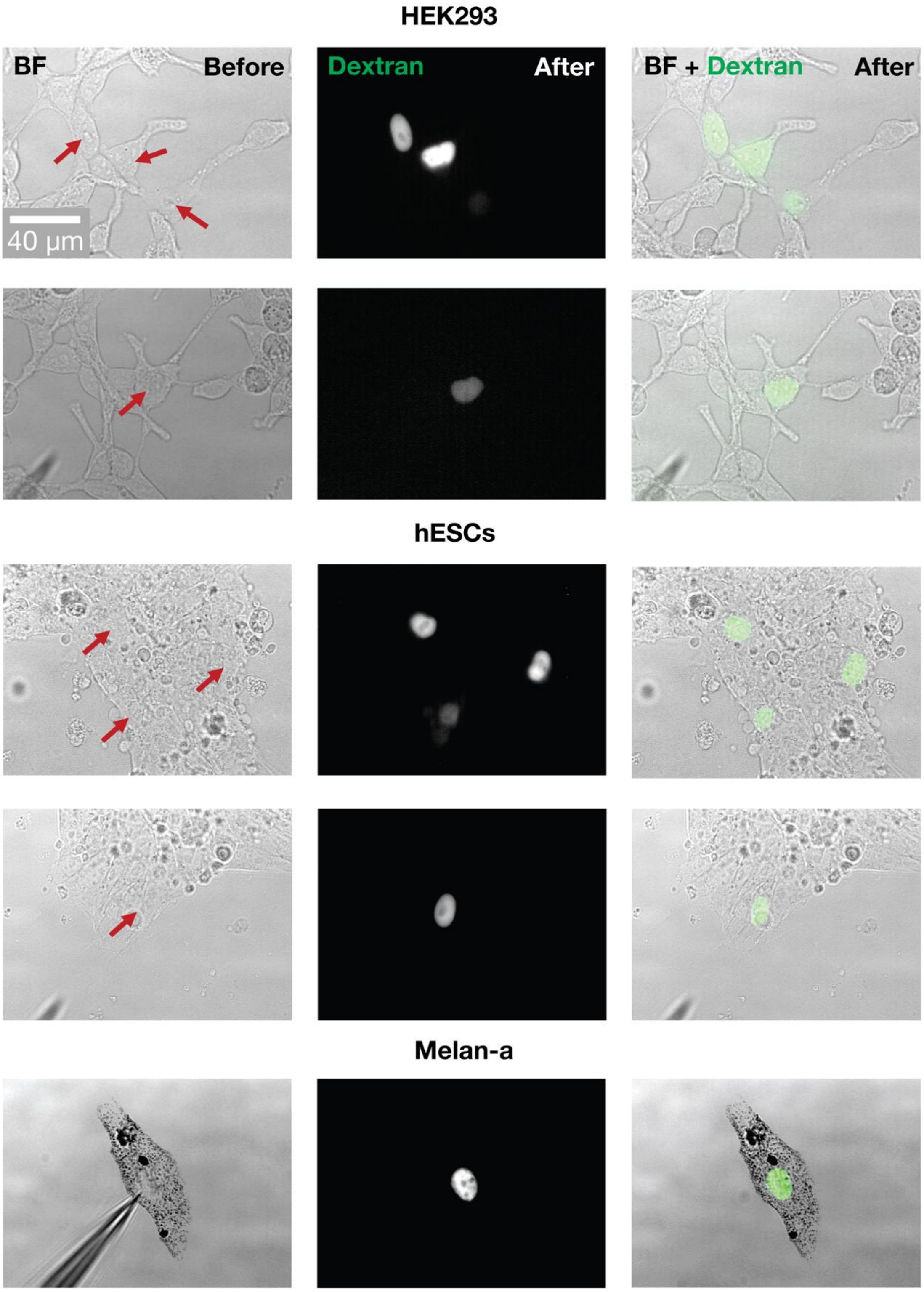

***Figure SF10**: Nuclear delivery in different cell lines. Human embryonic kidney (HEK), human embryonic stem cells (hESCs), mouse melanocytes (Melan-a).*

S4: Molecule properties as determinant of pulse shape

S4.1 Delivery of fluorescently tagged α-synuclein monomers into DRG neurons

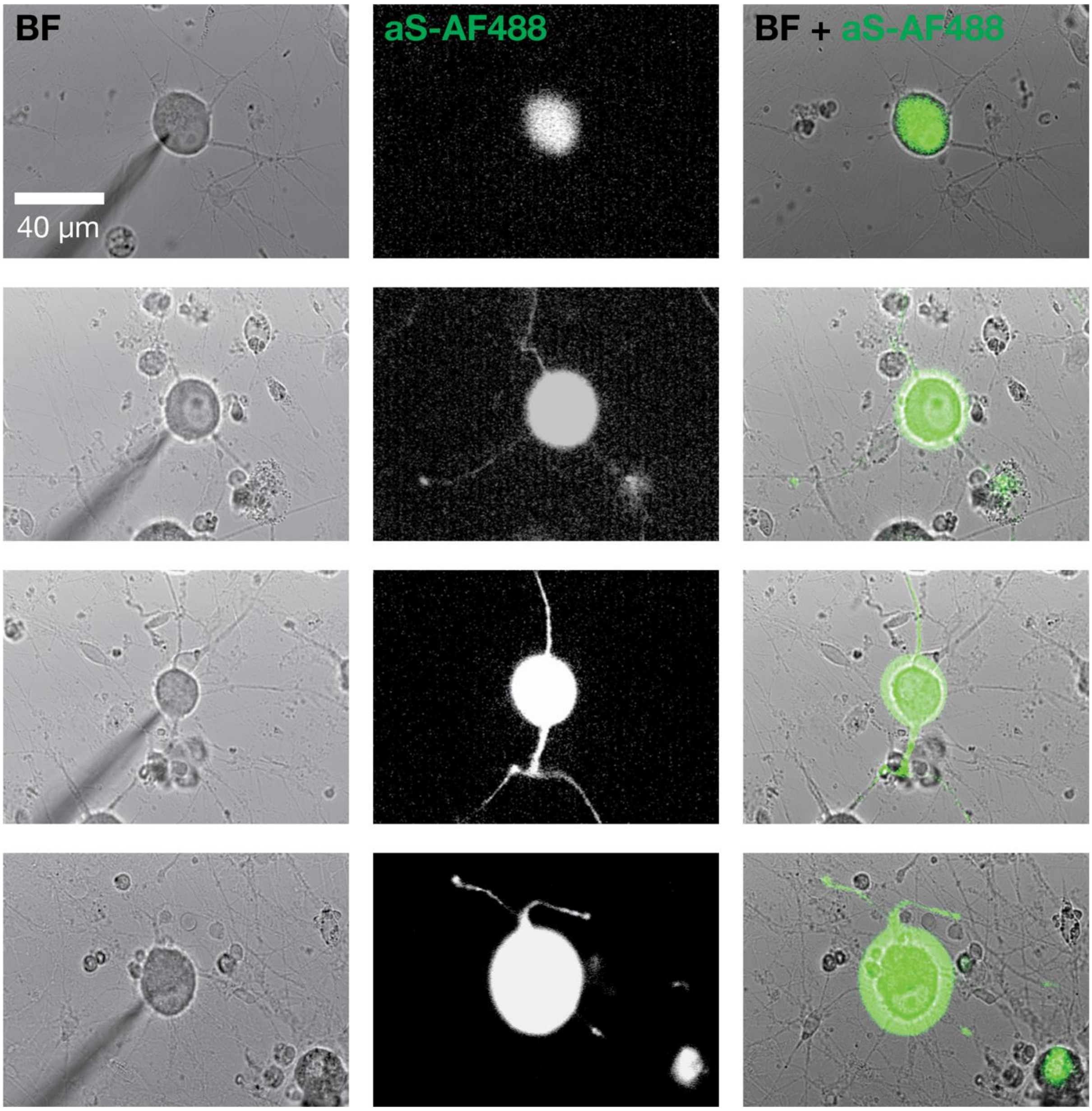

Figure SF11: *Delivery of Alexafluor488 tagged α-synuclein monomers into rat DRG neurons.*

S5: References

(1) Takahashi, Y.; Shevchuk, A. I.; Novak, P.; Zhang, Y.; Ebejer, N.; Macpherson, J. V.; Unwin, P. R.; Pollard, A. J.; Roy, D.; Clifford, C. A.; Shiku, H.; Matsue, T.; Klenerman, D.; Korchev, Y. E. Multifunctional Nanoprobes for Nanoscale Chemical Imaging and Localized Chemical Delivery at Surfaces and Interfaces. Angewandte Chemie International Edition 2011, 50 (41), 9638–9642. https://doi.org/10.1002/anie.201102796.

(2) Shevchuk, A.; Tokar, S.; Gopal, S.; Sanchez-Alonso, J. L.; Tarasov, A. I.; Vélez-Ortega, A. C.; Chiappini, C.; Rorsman, P.; Stevens, M. M.; Gorelik, J.; Frolenkov, G. I.; Klenerman, D.; Korchev, Y. E. Angular Approach Scanning Ion Conductance Microscopy. Biophysical Journal 2016, 110 (10), 2252–2265. https://doi.org/10.1016/j.bpj.2016.04.017.

(3) Novak, P.; Li, C.; Shevchuk, A. I.; Stepanyan, R.; Caldwell, M.; Hughes, S.; Smart, T. G.; Gorelik, J.; Ostanin, V. P.; Lab, M. J.; Moss, G. W. J.; Frolenkov, G. I.; Klenerman, D.; Korchev, Y. E. Nanoscale Live-Cell Imaging Using Hopping Probe Ion Conductance Microscopy. Nature Methods 2009, 6 (4), 279–281. https://doi.org/10.1038/nmeth.1306.

(4) Gialama, D.; Vadukul, D. M.; Thrush, R. J.; Radford, S. E.; Aprile, F. A. A Potent Sybody Selectively Inhibits α-Synuclein Amyloid Formation by Binding to the P1 Region. J. Med. Chem. 2024, 67 (12), 9857–9868. https://doi.org/10.1021/acs.jmedchem.3c02408.

(5) Perry, D.; Momotenko, D.; Lazenby, R. A.; Kang, M.; Unwin, P. R. Characterization of Nanopipettes. Analytical Chemistry 2016, 88 (10), 5523–5530. https://doi.org/10.1021/acs.analchem.6b01095.
